# Comparison of Oncologic Outcomes Between Incomplete Obstructive Colon Cancer and Non-Obstructive Colon Cancer by Tumor Location

**DOI:** 10.3389/fonc.2022.914299

**Published:** 2022-06-06

**Authors:** Ji Ha Lim, Woo Yong Lee, Seong Hyeon Yun, Hee Cheol Kim, Yong Beom Cho, Jung Wook Huh, Yoon Ah Park, Jung Kyong Shin

**Affiliations:** ^1^ Department of Surgery, Samsung Changwon Hospital, Sungkyunkwan University School of Medicine, Changwon, South Korea; ^2^ Department of Surgery, Samsung Medical Center, Sungkyunkwan University School of Medicine, Seoul, South Korea

**Keywords:** incomplete obstruction, colon cancer, stage, sidedness, survival

## Abstract

**Introduction:**

Obstruction in colon cancer is a well-known risk factor for worse oncologic outcomes. However, studies on differences in survival of patients with incomplete obstructive colon cancer (IOCC) by tumor location are insufficient. Thus, the aim of this study was to compare oncologic outcomes between IOCC and non-obstructive colon cancer (NOCC) according to tumor location.

**Methods:**

From January 2010 to December 2015, a total of 2,004 patients diagnosed with stage II or stage III colon adenocarcinoma who underwent elective colectomy were included (IOCC, *n* = 405; NOCC, *n* = 1,599). Incomplete obstruction was defined as a state in which colonoscopy could not pass through the cancer lesion but did not require emergent surgery, stent insertion, or stoma formation because the patient was asymptomatic without problem in bowel preparation. Kaplan–Meier method and log-rank tests were used to compare survival between IOCC and NOCC. Multivariable analysis was performed to determine which factors affected survivals.

**Results:**

Stage III IOCC patients showed significantly lower overall survival (OS) and recurrence-free survival (RFS). Stage II IOCC patients and stage III NOCC patients had similar survival curves. IOCC patients with tumors on the right side showed worse OS than other patients. In multivariable analysis, incomplete obstruction was an independent risk factor for worse OS and RFS in all stages. Tumor located at the right side in stage III was an independent risk factor for RFS (HR: 1.40, *p* = 0.030).

**Conclusions:**

Patients with IOCC showed significantly worse survival outcomes than those with NOCC. Stage II IOCC patients and stage III NOCC patients showed similar survival. Patients with stage III IOCC located at the right side showed significantly worse oncologic outcomes than those located at the left side. These results confirm that prognosis is different depending on the presence of incomplete obstruction and the location of the tumor, even in the same stage.

## Introduction

Obstruction is presented in approximately 10% to 29% of colorectal cancer cases. It is the most common cause of large intestinal obstruction (1–3). Patients with acute complete obstructive colon cancer (OCC) need emergent colectomy with or without anastomosis, colonic stent insertion, or primary stoma formation. Surgery under this emergent condition is associated with increased morbidity and mortality, affecting survival outcomes (4–6). Moreover, complete obstruction by colorectal cancer is associated with worse survival (3, 7–9). However, studies so far have only analyzed or included an acute obstructive condition that needs instant treatment. Studies on survival of patients with incomplete obstructive colon cancer (IOCC), which does not need instant treatment for resolving obstruction, are insufficient. Studies comparing oncologic outcomes between right-sided and left-sided OCC are also scarce. Some studies have reported that right-sided OCC has a worse prognosis than left-sided OCC (10–12). However, other studies have shown no differences in oncologic outcome between left-side and right-side OCCs (13, 14). Accordingly, the aim of this study was to compare oncologic outcomes of IOCC patients with those of non-obstructive colon cancer (NOCC) patients. Differences in outcomes between right-sided and left-sided IOCC were also determined.

## Materials and Methods

**Figure 1:**
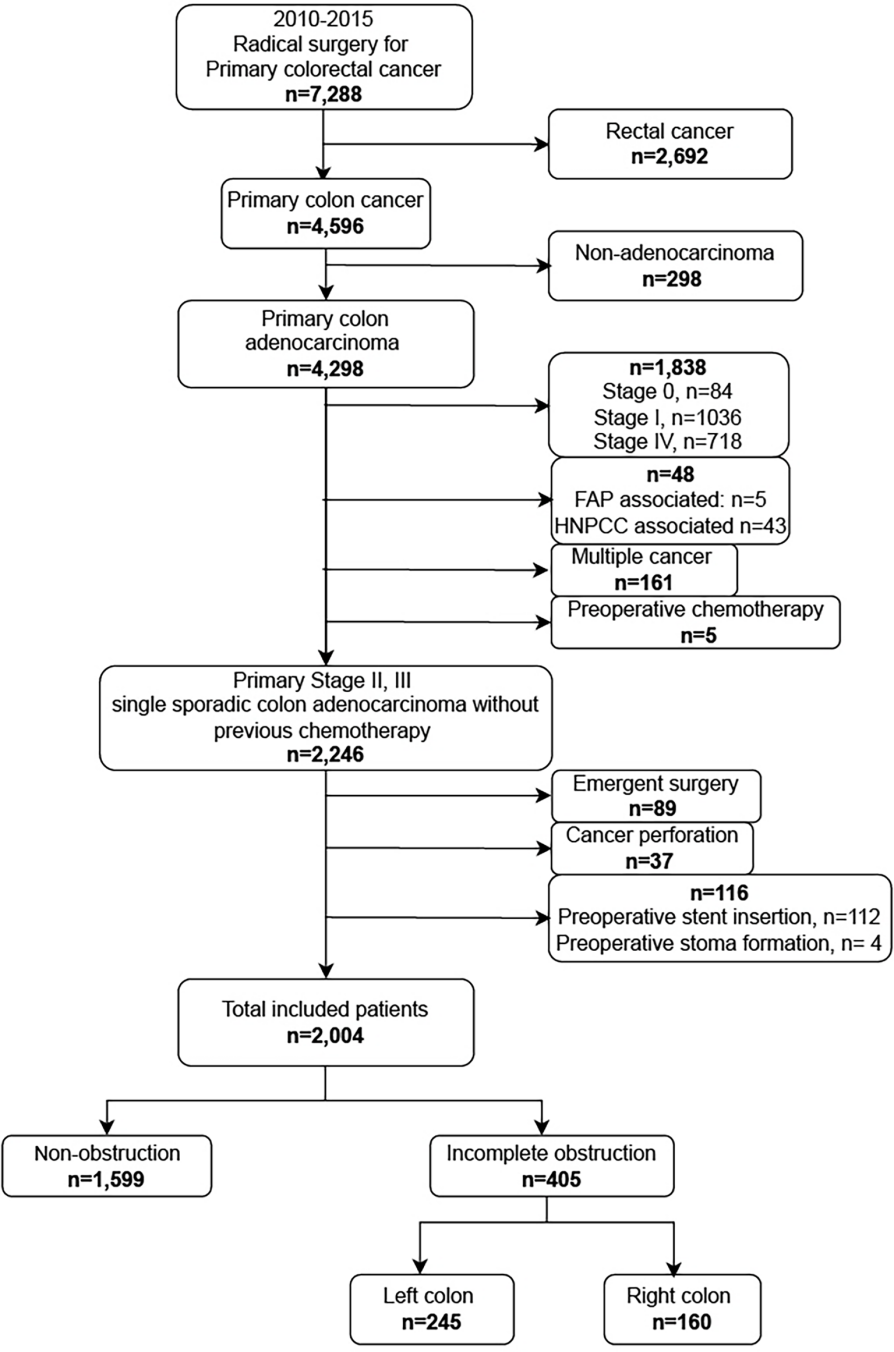
Flowchart of the study indicating the inclusion and exclusion criteria.

From January 2010 to December 2015, 7,288 patients who underwent surgery for primary colorectal cancer in Samsung Medical Center (SMC) were identified. Patients who were diagnosed with rectal cancer; who were not diagnosed with adenocarcinoma, stage II and III; who had hereditary colon diseases and multiple colon cancer; who underwent preoperative chemotherapy and emergent surgery; who had cancer peroration; and who had colonic stent placed or formed a stoma for obstructive colon cancer before radical colectomy were excluded. A total of 2,004 patients were included and analyzed in this study (**Figure 1**).

Information on age, sex, body mass index (BMI), American Society of Anesthesiologists (ASA) score, preoperative erythrocyte sedimentation rate (ESR), C-reactive protein (CRP), carcinoembryonic antigen (CEA), carbohydrate antigen 19-9 (CA 19-9) level, and tissue samples after colectomy was collected. All patients underwent colectomy without stoma formation. Several pathologists evaluated all specimens. Stages were classified according to the American Joint Committee on Cancer (AJCC) 8th guidelines (15). Data on any postoperative complications were collected. Among them, data on Clavien–Dindo classification (CDC) (16, 17) grade 3 (needing surgical, endoscopic, or radiological intervention) or higher were collected with anastomosis leakage data separately. Anastomosis leakage was defined as any condition showing the presence of an abscess around the anastomosis or anastomosis site dehiscence identified on imaging examination. Readmission data within 30 days after surgery were also collected.

Adjuvant chemotherapy was performed based on the National Comprehensive Cancer Network (NCCN) guidelines. If patients refused, routine follow-up was performed. Follow-up examinations included serum CEA level, abdomen and chest CT, and colonoscopy. Usually, follow-up started 2 weeks after the discharge date and was performed every 3 months for the first 3 years and every 6 months until 5 years after that. However, if patients wanted to revisit after 5 years of follow-up, additional follow-up was performed. Moreover, if recurrence was suspected, follow-up could be shortened and further evaluation was performed.

Group was initially divided into IOCC and NOCC. Incomplete obstruction was defined as a state in which colonoscopy could not pass through the cancer lesion but did not require emergent surgery, stent insertion, or stoma formation because the patient was asymptomatic without problem in bowel preparation. Subgroup analysis was performed by tumor location (left colon vs. right colon). The ascending colon to the mid-transverse colon was included in the right colon. The distal transverse colon to the rectosigmoid colon was included in the left colon.

All statistical analyses were performed using SPSS version 27.0 (SPSS Inc., Chicago, IL, USA). A *p*-value of less than 0.05 was considered statistically significant. Except for the preoperative CRP (missing value of 12.7%) and ESR (missing value of 11.3%), all other data missing values were less than 2%, and any missing data were omitted and the remaining data were analyzed. Comparison of baseline characteristics was performed using *χ* ^2^ test or Fisher’s exact test for categorical variables. Continuous variables were compared using Student’s *t*-test and Mann–Whitney test after checking normality of data with the Shapiro–Wilk test. Survival was analyzed with the Kaplan–Meier method and log-rank test. Univariable and multivariable analyses were performed using Cox proportional-hazards (PH) regression analysis to determine which factors affected survival. Variables that showed significance in univariable Cox PH regression analysis were entered into a multivariable Cox PH regression analysis using the backward elimination method. This study was approved by the Institutional Review Board of SMC (Approval number: SMC 2021-06-041).

## Results

A total of 2,004 patients pathologically diagnosed with stage II or III colon cancer who underwent elective colectomy for IOCC or NOCC from 2010 to 2015 were included in this study (IOCC, *n* = 405; NOCC, *n* = 1,599). Baseline characteristics of subjects with IOCC or NOCC are shown in **Table 1**. Preoperative median ESR and CRP levels were higher in the IOCC group [ESR: 34 (range, 2–120) mm/h vs. 25 (range, 2–120) mm/h, *p* < 0.001; CRP: 0.28 (range, 0.03–13.53) mg/dl vs. 0.12 (range, 0.02–34.12) mg/dl, *p* < 0.001]. More patients showed elevated CEA (over 5 ng/ml) and CA 19-9 (over 37 ng/ml) levels in the IOCC group (elevated CEA: 30.4% vs. 16.8%, *p* < 0.001; elevated CA: 19-9, 15.3% vs. 7.8%, *p* < 0.001). The IOCC group had more open surgery cases (23.2% vs. 11.5%, *p* < 0.001), longer mean operative time (150.79 ± 55.32 min vs. 143.63 ± 46.70 min, *p* = 0.017), and more blood loss (115.55 ± 108.00 ml vs. 97.48 ± 82.54 ml, *p* = 0.002). Although the proportion of left colon cancer was higher in the IOCC group than in the NOCC group, the difference was not statistically significant (60.5% vs. 57.5%, *p* = 0.281, **Table 1**).

**Table 1:** Baseline characteristics of incomplete obstructive colon cancer (IOCC) and non-obstructive colon cancer (NOCC).

	Total (*n* = 2,004)	IOCC (*n* = 405)	NOCC (*n* = 1599)	*p*-value
Age, years				
<60 years ≥60 years	900 (44.9%) 1,104 (55.1%)	170 (42.0%) 235 (58.0%)	730 (45.7%) 869 (78.7%)	0.184
Sex				
Male Female	1,098 (54.8%) 906 (45.2%)	214 (52.8%) 191 (47.2%)	884 (55.3%) 715 (44.7%)	0.377
BMI, (range), kg/m^2^	23.46 (15.00–44.06)	23.00 (15.02–36.00)	23.72 (15.00–44.06)	<0.001
ASA score				
1 2 3	710 (35.4%) 1,216 (60.7%) 78 (3.9%)	152 (37.5%) 238 (58.8%) 15 (3.7%)	558 (34.9%) 978 (61.2%) 62 (3.9%)	0.706
Preoperative ESR (range), mm/h	26 (2–120)	34 (2–120)	25 (2–120)	<0.001
Preoperative CRP (range), mg/dl	0.14 (0.02–34.12)	0.28 (0.03–13.53)	0.12 (0.02–34.12)	<0.001
Preoperative CEA ≥5 ng/ml	391 (19.5%)	123 (30.4%)	268 (16.8%)	<0.001
Preoperative CA 19-9 ≥37 ng/ml	187(9.3%)	62 (15.3%)	125 (7.8%)	<0.001
Tumor location				
Right Left	839 (41.9%) 1,165 (58.1%)	160 (39.5%) 245 (60.5%)	679 (42.5%) 920 (57.5%)	0.281
Surgical technique				
Open MIS*	278 (13.9%) 1,726 (86.1%)	94 (23.2%) 311 (76.8%)	184 (11.5%) 1,415 (88.5%)	<0.001
Operative time, mean ± SD, min	145.07 ± 48.64	150.79 ± 55.32	143.63 ± 46.70	0.017
Amount of blood loss, mean ± SD, ml	01.13 ± 88.54	115.55 ± 108.00	97.48 ± 82.54	0.002

SD, standard deviation; BMI, body mass index; ASA, American Society of Anesthesiologists; ESR, erythrocyte sedimentation rate; CRP, C-reactive protein; CEA, carcinoembryonic antigen; CA 19-9, carbohydrate antigen 19-9; MIS, minimally invasive surgery.

*This includes hand-assisted laparoscopy, total laparoscopy, and robotic surgery.

In pathologic results, patients with IOCC showed bigger tumor size (5.96 ± 2.12 cm vs. 4.54 ± 2.09 cm, *p* < 0.001), more serosal exposure tumors (T4: 27.2% vs. 14.9%, *p* < 0.001), and more moderately (MD) and poorly differentiated (PD) adenocarcinomas (MD+PD: 83.0% vs. 76.5%, *p* = 0.006). However, there was no statistical significance in nodal status (*p* = 0.139) or tumor-nodal-metastasis (TNM) stage (*p* = 0.713) between the two groups. Lymphatic invasion and high microsatellite instability (MSI) status were not significantly different between IOCC and NOCC groups (both *p* > 0.05), but perineural invasion (40.7% vs. 30.3%, *p* < 0.001), vascular invasion (18.3% vs. 13.1%, *p* = 0.007), and positive tumor budding (62.0% vs. 55.7%, *p* = 0.022) were more common in IOCC than in NOCC. Any morbidity (18.8% vs. 14%, *p* = 0.017) and morbidity ≥ CDC grade 3 (6.7% vs. 3.4%, *p* = 0.003) were more common in IOCC. There was no significant difference in anastomotic leakage (*p* = 0.596) between the two groups. Furthermore, there was no significant difference in the proportion of patients receiving adjuvant chemotherapy (overall: 67.9% vs. 68.3%; stage II: 43.6% vs. 40.5%; stage III: 87.2% vs. 91.2%, all *p* > 0.05). However, the recurrence rate was higher in the IOCC group (20.2% vs. 10.4%, *p* < 0.001), although there was no statistically significant difference in the recurrence pattern (*p* = 0.621, **Table 2**).

**Table 2:** Postoperative outcomes of incomplete obstructive colon cancer (IOCC) and non-obstructive colon cancer (NOCC).

	Total (*n* = 2,004)	IOCC (*n* = 405)	NOCC (*n* = 1,599)	*p*-value
Tumor size, mean ± SD, cm	4.83 ± 2.17	5.96 ± 2.12	4.54 ± 2.09	<0.001
Cell differentiation				
WD MD/PD	445 (22.2%) 1,559 (77.8%)	69 (17.0%) 336 (83.0%)	376 (23.5%) 1,223 (76.5%)	0.006
TNM stage				
Stage II Stage III	902 (45.0%) 1,102 (55.0%)	179 (44.2%) 226 (55.8%)	723 (45.2%) 876 (54.8%)	0.713
T classification				
T1/T2 T3 T4	145 (7.2%) 1,510 (75.4%) 349 (17.4%)	3 (0.7%) 292 (72.1%) 110 (27.2%)	142 (8.9%) 1218 (76.2%) 239 (14.9%)	<0.001
N classification				
N0 N1 N2	902 (45.0%) 784 (39.1%) 318 (15.9%)	179 (44.2%) 149 (36.8%) 77 (19.0%)	723 (45.2%) 635 (39.7%) 241 (15.1%)	0.139
Harvested lymph node (range), n	21 (2–74)	23 (5–74)	21 (2–24)	<0.001
Lymphatic invasion	793 (39.6%)	172 (42.6%)	621 (38.9%)	0.176
Perineural invasion	646 (32.4%)	165 (40.7%)	481 (30.3%)	<0.001
Vascular invasion	282 (14.2%)	74 (18.3%)	208 (13.1%)	0.007
Positive tumor budding	1,137 (56.9%)	251 (62.0%)	886 (55.7%)	0.022
MSI-H	164 (8.3%)	39 (9.7%)	125 (7.9%)	0.243
Postoperative complication CDC ≥ grade 3 Anastomotic leakage	300 (15.0%) 82 (4.1%) 29 (1.5%)	76 (18.8%) 27 (6.7%) 7 (1.7%)	224(14.0%) 55 (3.4%) 22 (1.4%)	0.017 0.003 0.596
Length of stay, mean ± SD, day	7.36 ± 8.93	7.77 ± 4.49	7.25 ± 9.73	0.295
Readmission	70 (3.5%)	17 (4.2%)	53 (3.3%)	0.387
Adjuvant chemotherapy Stage II Stage III	1,367 (68.2%) 371 (41.1%) 996 (90.4%)	275 (67.9%) 78 (43.6%) 197 (87.2%)	1,092 (68.3%) 293 (40.5%) 799 (91.2%)	0.880 0.458 0.066
Recurrence Systemic Local Combined	248 (12.4%) 22 (8.9%) 216 (87.1%) 10 (4.0%)	82 (20.2%) 9 (11.0%) 69 (84.1%) 4 (4.9%)	166 (10.4%) 13 (7.8%) 147 (88.6%) 6 (3.6%)	<0.001 0.621

SD, standard deviation; WD, well-differentiated; MD, moderately differentiated; PD, poorly differentiated; MSI-H, microsatellite instability-high; CDC, Clavien–Dindo classification.

### Sub-Analysis of IOCC by Tumor Location

Of 405 IOCC patients, 245 had left colon cancer and 160 had right colon cancer. Preoperative ESR and CRP were higher in the right colon cancer group [ESR: 40 (range, 3–120) mm/h vs. 28.5 (range, 2–120) mm/h, *p* < 0.001; CRP: 0.41 mg/dl vs. 0.23 mg/dl, *p* = 0.023]. More patients showed elevated CA 19-9 level (over 37 ng/ml) in the right IOCC group (23.8% vs. 9.8%, *p* < 0.001). However, the proportion of patients who showed elevated CEA level (over 5 ng/ml) did not differ between left and right colon cancer groups (*p* = 0.216, **Table 3**). Results of comparing postoperative outcomes between right and left IOCC groups are shown in **Table 4**. Right colon cancer showed bigger tumor size (6.61 ± 2.36 cm vs. 5.54 ± 1.83 cm, *p* < 0.001), more T4 cancer (35.0% vs. 22.0%, *p* = 0.006), more MSI high tumor (18.4% vs. 4.1%, *p* < 0.001), and higher recurrence rate (26.3% vs. 16.3%, *p* = 0.015). However, there were no significant differences in nodal status, stage, or risk factors such as lymphatic invasion, vascular invasion, perineural invasion, or positive tumor budding (all *p* > 0.05, **Table 4**).

**Table 3:** Baseline characteristics of incomplete obstructive colon cancer (IOCC) by tumor location.

	Left colon (*n* = 245)	Right colon (*n* = 160)	*p*-value
Age, years			
<60 years ≥60 years	111 (45.3%) 134 (54.7%)	59 (36.9%) 101 (63.1%)	0.093
Sex			
Male Female	135 (55.1%) 110 (44.9%)	79 (49.4%) 81 (50.6%)	0.259
BMI, median (range), kg/m^2^	23 (15.4–36)	23 (15.0–32.7)	0.633
ASA score			
1 2 3	100 (40.8%) 135 (55.1%) 10 (4.1%)	52 (32.5%) 103 (64.4%) 5 (3.1%)	0.179
Preoperative ESR (range), mm/h	28.5 (2–120)	40 (3–120)	<0.001
Preoperative CRP (range), mg/dl	0.23 (0.03–13.53)	0.41 (0.03–9.55)	0.023
Preoperative CEA ≥5 ng/ml	80 (32.7%)	43 (26.9%)	0.216
Preoperative CA19-9 ≥37 ng/ml	24 (9.8%)	38 (23.8%)	<0.001
Surgical technique			
Open MIS*	52 (21.2%) 193 (78.8%)	42 (26.3%) 118 (73.8%)	0.242
Operative time, mean ± SD, min	145.04 ± 48.76	159.61 ± 63.24	0.009
Amount of blood loss, mean ± SD, ml	108.24 ± 87.82	126.47 ± 132.12	0.127

SD, standard deviation; BMI, body mass index; ASA, American Society of Anesthesiologists; ESR, erythrocyte sedimentation rate; CRP, C-reactive protein; CEA, carcinoembryonic antigen; CA 19-9, carbohydrate antigen 19-9; MIS, minimally invasive surgery.

*This includes hand-assisted laparoscopy, total laparoscopy, and robotic surgery.

**Table 4:** Postoperative outcomes of incomplete obstructive colon cancer (IOCC) by tumor location.

	Left colon (*n* = 245)	Right colon (*n* = 160)	*p*-value
Tumor size, mean ± SD, cm	5.54 ± 1.83	6.61 ± 2.36	<0.001
Cell type			
WD MD/PD	38 (15.5%) 207 (84.5%)	31 (19.4%) 129 (80.6%)	0.312
TNM stage			
Stage II Stage III	108 (44.1%) 137 (55.9%)	71 (44.4%) 89 (55.6%)	0.954
T classification			
T1/T2 T3 T4	3 (1.2%) 188 (76.7%) 54 (22.0%)	0 (0%) 104 (65.0%) 56 (35.0%)	0.006
N classification			
N0 N1 N2	108 (44.1%) 90 (36.7%) 47 (19.2%)	71 (44.4%) 59 (36.9%) 30 (18.8%)	0.994
Harvested lymph node (range), *n*	21 (5–74)	27 (7–56)	<0.001
Lymphatic invasion	101 (41.2%)	71 (44.7%)	0.496
Perineural invasion	108 (44.1%)	57 (35.6%)	0.090
Vascular invasion	42 (17.1%)	32 (20.1%)	0.449
Positive tumor budding	144(58.8%)	107(66.9%)	0.101
MSI-H	10 (4.1%)	29 (18.4%)	<0.001
Postoperative complication CDC ≥ grade 3 Anastomotic leakage	42 (17.1%) 15 (6.1%) 5 (2.0%)	34 (21.3%) 12 (7.5%) 2 (1.3%)	0.301 0.587 0.708
Length of stay, mean ± SD, day	7.59 ± 3.88	8.05 ± 5.30	0.312
Readmission	9 (3.7%)	8 (5.0%)	0.515
Adjuvant chemotherapy Stage II Stage III	169 (69.0%) 45 (41.7%) 124 (90.5%)	106 (66.3%) 33 (46.5%) 73 (82.0%)	0.565 0.630 0.097
Recurrence Systemic Local Combined	40 (16.3%) 4 (10.0%) 35 (87.5%) 1 (2.5%)	42 (26.3%) 5 (11.9%) 34 (81.0%) 3 (7.1%)	0.015 0.801

SD, standard deviation; WD, well-differentiated; MD, moderately differentiated; PD, poorly differentiated; MSI-H, microsatellite instability-high; CDC, Clavien–Dindo classification.

### Comparison of Overall Survival Between IOCC and NOCC by Stage and Tumor Location

**Figure 2:**
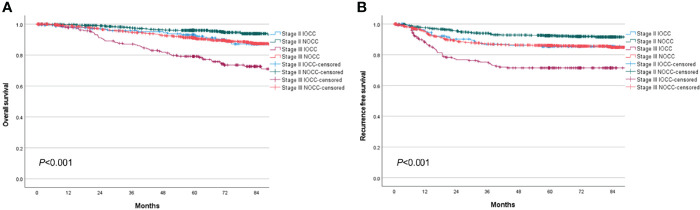
Overall survival and recurrence-free survival between IOCC and NOCC by stage. **(A)** Overall survival by obstruction status and stage. **(B)** Recurrence-free survival by obstruction status and stage.

**Figure 3:**
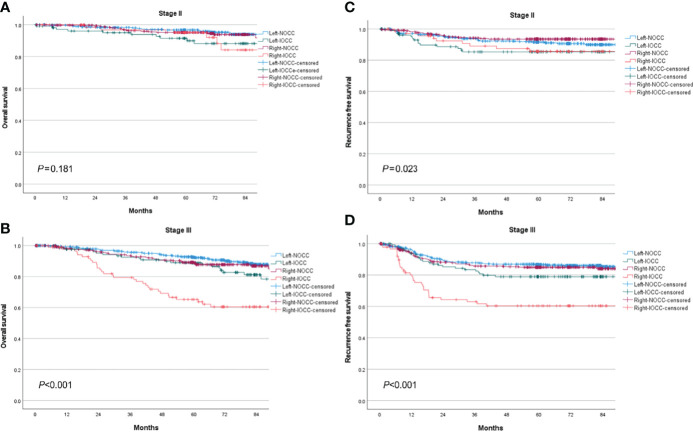
Overall survival and recurrence-free survival between stage II and stage III IOCC and NOCC by tumor location. **(A)** Overall survival of stage II colon cancer by obstruction status and tumor location. **(B)** Overall survival of stage III colon cancer by obstruction status and tumor location. **(C)** Recurrence-free survival of stage II colon cancer by obstruction status and tumor location. **(D)** Recurrence-free survival of stage III colon cancer by obstruction status and tumor location.

The total median follow-up period was 68.6 (range, 0.1–128.8) months, and follow-up loss occurred in 18.6% of patients. Stage III IOCC patients showed significantly lower survival, while stage II IOCC patients and stage III NOCC patients had similar survival curves (5-year OS: 93.0% for stage II IOCC, 96.0% for stage II NOCC, 79.2% for stage III IOCC, 91.2% for stage III NOCC, *p* < 0.001, **Figure 2A**). In a sub-analysis by tumor location, there was no significant difference in survival for stage II (*p* = 0.181, **Figure 3A**). However, for stage III, right IOCC patients showed worse overall survival than other patients (5-year OS: 65.2% for right IOCC, 88.9% for left IOCC, 89.1% for right NOCC, 92.5% for left NOCC, *p* < 0.001, **Figure 3B**).

In multivariable Cox regression analysis of stage II colon cancer, incomplete obstruction was an independent risk factor for worse OS (HR: 1.86, 95% CI: 1.03–3.37, *p* = 0.040). Elevated CEA levels and postoperative complication were also independent risk factors (CEA level, HR: 2.02, 95% CI: 1.10–3.70, *p* = 0.023; morbidity, HR: 2.73, 95% CI: 1.54–4.83, *p* = 0.001). Age over 60 years was also a strong risk factor (HR: 12.87, 95% CI: 4.00–41.44, *p* < 0.001).

For stage III, incomplete obstruction was also an independent risk factor for worse OS (HR: 1.51, 95% CI: 1.06–2.14, *p* = 0.022). Age over 60 years (HR: 2.79, 95% CI: 1.87–4.16, *p* < 0.001), elevated CA 19-9 level (HR: 2.23, 95% CI: 1.51–3.31, *p* < 0.001), N2 nodal status (HR: 1.48, 95% CI: 1.03–2.13, *p* = 0.035), moderately differentiated feature (HR: 2.62, 95% CI: 1.21–5.71, *p* = 0.015), poorly differentiated feature (HR: 2.63, 95% CI: 1.02–6.78, *p* = 0.045), vascular invasion (HR: 1.81, 95% CI: 1.25–2.64, *p* = 0.002), and not undergoing or completing adjuvant chemotherapy (HR: 3.82, 95% CI: 2.53–5.77, *p* < 0.001) were other risk factors of overall survival (**Table 6**). However, tumor location was not an independent factor affecting overall survival for any stage (**Tables 5**, **6**).

**Table 5:** Univariable and multivariable Cox regression analysis of overall survival and recurrence-free survival of stage II colon cancer.

			Overall survival						Recurrence-free survival					
			Univariable analysis			Multivariable analysis			Univariable analysis			Multivariable analysis		
	Reference		HR	95% CI	*p*-value	HR	95% CI	*p*-value	HR	95% CI	*p*-value	HR	95% CI	*p*-value
**Incomplete obstruction**	N	Y	1.91	1.06–3.45	0.032	1.86	1.03–3.37	0.040	1.99	1.22–3.22	0.005	1.84	1.13–2.99	0.014
**Tumor location**	Lt.	Rt.	1.03	0.59–1.78	0.926				0.73	0.46–1.15	0.176			
**Age**	<60	≥60	13.69	4.26–43.99	<0.001	12.87	4.00–41.44	< 0.001	1.07	0.68–1.69	0.759			
**Sex**	Female	Male	0.80	0.46–1.39	0.435				1.26	0.79–2.00	0.336			
**Surgical technique**	Open	MIS	0.40	0.22–0.73	0.003	0.69	0.36–1.33	0.270	0.64	0.36–1.13	0.122			
**Preoperative CEA**	<5	≥5	2.21	1.21–4.04	0.010	2.02	1.10–3.70	0.023	2.06	1.25–3.40	0.005	1.81	1.09–3.01	0.021
**Preoperative CA19-9**	<37	≥37	2.21	0.97–4.91	0.051				1.43	0.66–3.11	0.369			
**Postoperative complication**	N	Y	3.41	1.93–6.03	<0.001	2.73	1.54–4.83	0.001	1.24	0.68–2.25	0.484			
**Tumor size**			1.03	0.91–1.16	0.626				0.99	0.89–1.10	0.791			
**Pathologic T classification**	T3	T4	1.78	0.86–3.65	0.118				2.53	1.47–4.33	0.001	2.17	1.26–3.75	0.005
**Harvested LNs**	<12	≥12	1.79	0.87–3.68	0.114				0.89	0.41–1.94	0.768			
**Cell differentiation**	WD	MD	0.94	0.28–3.18	0.915				1.50	0.88–2.58	0.140			
		PD	1.07	0.21–2.20	0.512				0.37	0.05–2.75	0.329			
**Lymphatic invasion**	N	Y	1.52	0.78–2.96	0.223				1.13	0.63–2.02	0.682			
**Perineural invasion**	N	Y	0.87	0.42–1.79	0.704				1.29	0.80–1.98	0.318			
**Vascular invasion**	N	Y	1.02	0.25–4.22	0.973				1.05	0.33–3.32	0.941			
**Tumor budding**	N	Y	1.28	0.74–2.23	0.379				1.26	0.80–1.98	0.318			
**MSI status**	High	Low/MSS	3.24	0.79–13.35	0.103				10.59	1.47–76.16	0.019	10.17	1.41–73.18	0.021
**Adjuvant chemotherapy completion**	Y	N	2.40	1.25–4.58	0.008	1.64	0.85–3.16	0.137	1.04	0.66–1.64	0.873			

MIS, minimally invasive surgery; CEA, carcinoembryonic antigen; CA 19-9, carbohydrate antigen 19-9; WD, well-differentiated; MD, moderately differentiated; PD, poorly differentiated; MSI, microsatellite instability; MSS, microsatellite stable.

**Table 6:** Univariable and multivariable Cox regression analysis of overall survival and recurrence-free survival of stage III colon cancer.

			Overall survival						Recurrence-free survival					
			Univariable analysis			Multivariable analysis			Univariable analysis			Multivariable analysis		
	Reference		HR	95% CI	*p*-value	HR	95% CI	*p*-value	HR	95% CI	*p*-value	HR	95% CI	*p*-value
**Incomplete obstruction**	N	Y	2.45	1.74–3.44	<0.001	1.51	1.06–2.14	0.022	2.15	1.57–2.95	<0.001	1.69	1.22–2.34	0.002
**Tumor location**	Lt.	Rt.	1.68	1.19–2.30	0.003	1.05	0.72–1.51	0.815	1.42	1.06–1.92	0.021	1.40	1.03–1.91	0.030
**Age**	<60	≥60	3.46	2.36–5.07	<0.001	2.79	1.87–4.16	<0.001	1.46	1.08–1.98	0.014	1.21	0.87–1.67	0.253
**Sex**	Female	Male	1.17	0.84–1.63	0.351				0.75	0.55–1.01	0.055			
**Surgical technique**	Open	MIS	0.40	0.28–0.58	<0.001	0.64	0.43–0.93	0.021	0.65	0.44–0.95	0.027	0.78	0.52–1.15	0.208
**Preoperative CEA**	<5	≥5	1.80	1.26–2.56	0.001	0.95	0.64–1.40	0.780	1.25	0.88–1.76	0.216			
**Preoperative CA19-9**	<37	≥37	3.02	2.07–4.41	<0.001	2.23	1.51–3.31	<0.001	2.47	1.71–3.56	<0.001	1.92	1.31–2.81	0.001
**Postoperative complication**	N	Y	1.40	0.92–2.11	0.114				1.13	0.75–1.69	0.565			
**Tumor size**			1.15	1.07–1.23	<0.001	1.07	0.97–1.17	0.185	1.07	1.00–1.14	0.051			
**Pathologic T category**	T1	T2	2.23	0.26–19.08	0.464	1.16	0.13–9.93	0.895	4.55	0.58–35.51	0.149			
		T3	4.14	0.57–29.83	0.159	1.42	0.19–10.39	0.729	5.57	0.78–39.98	0.088			
		T4	15.51	2.16–111.58	0.006	3.58	0.48–26.46	0.211	15.86	2.20–114.13	0.006			
**Pathologic N category**	N1	N2	2.12	1.53–2.95	<0.001	1.48	1.03–2.13	0.035	1.97	1.45–2.66	<0.001	1.60	1.16–2.21	0.004
**Harvested LNs**	<12	≥12	1.27	0.79–2.04	0.320				0.93	0.57–1.52	0.785			
**Cell differentiation**	WD	MD	3.96	1.85–8.48	<0.001	2.62	1.21–5.71	0.015	1.74	1.06–2.84	0.028	1.38	0.81–2.36	0.241
		PD	6.57	2.68–16.12	<0.001	2.63	1.02–6.78	0.045	3.04	1.58–5.85	0.001	1.82	0.89–3.72	0.103
**Lymphatic invasion**	N	Y	1.42	1.00–2.00	0.048	0.96	0.66–1.40	0.833	1.29	0.95–1.76	0.105			
**Perineural invasion**	N	Y	1.63	1.18–2.27	0.003	1.26	0.87–1.82	0.216	1.81	1.34–2.45	<0.001	1.67	1.22–2.27	0.001
**Vascular invasion**	N	Y	1.99	1.41–2.80	<0.001	1.81	1.25–2.64	0.002	2.08	1.52–2.84	<0.001	1.66	1.19–2.31	0.003
**Tumor budding**	N	Y	1.62	1.11–2.37	0.012	1.00	0.67–1.51	0.983	1.58	1.12–2.23	0.009	1.17	0.82–1.68	0.394
**MSI status**	High	Low/MSS	0.96	0.47–1.95	0.902				1.12	0.55–2.27	0.759			
**Adjuvant chemotherapy completion**	Y	N	5.50	3.76–8.04	<0.001	3.82	2.53–5.77	<0.001	2.74	1.85–4.07	<0.001	3.06	2.05–4.58	<0.001

MIS, minimally invasive surgery; CEA, carcinoembryonic antigen; CA 19-9, carbohydrate antigen 19-9; WD, well-differentiated; MD, moderately differentiated; PD, poorly differentiated; MSI, microsatellite instability; MSS, microsatellite stable.

### Comparison of Recurrence-Free Survival between IOCC and NOCC by Stage and Tumor Location

The RFS curve showed a similar pattern to the OS curve. Stage III IOCC patients had significantly lower RFS than other patients, while similar survival was found for stage II IOCC and stage III NOCC (5-year RFS: 85.4% for stage II IOCC, 92.5% for stage II NOCC, 71.5% for stage III IOCC, 86.0% for stage III NOCC, *p* < 0.001, **Figure 2B**). In a sub-analysis by tumor location, there was a difference in RFS according to the presence of incomplete obstruction, not the location of the tumor, in patients with stage II cancer (5-year RFS: 85.6% for right IOCC, 85.3% for left IOCC, 93.5% for right NOCC, 91.5% for left NOCC, *p* = 0.023, **Figure 3C**). For stage III, right IOCC patients showed significantly worse RFS (5-year RFS: 60.4% for right IOCC, 78.9% for left IOCC, 85.0% for right NOCC, and 86.7% for left NOCC, *p* < 0.001, **Figure 3D**).

In multivariable Cox regression analysis of stage II colon cancer, incomplete obstruction was an independent risk factor for worse RFS (HR: 1.84, 95% CI: 1.13–2.99, *p* = 0.014). Elevated CEA level (HR: 1.81, 95% CI: 1.09–3.01, *p* = 0.021), T4 adenocarcinoma (HR: 2.17, 95% CI: 1.26–3.75, *p* = 0.005), and MSI low/microsatellite stable (MSS) tumors (HR: 10.17, 95% CI: 1.41–73.18, *p* = 0.021) were also independent risk factors for worse RFS. However, tumor location did not independently affect RFS (**Table 5**).

Incomplete obstruction was also an independent risk factor for worse RFS in stage III (HR: 1.69, 95% CI: 1.22–2.34, *p* = 0.002). Elevated CA 19-9 level (HR: 1.92, 95% CI: 1.31–2.81, *p* = 0.001), N2 nodal status (HR: 1.60, 95% CI: 1.16–2.21, *p* = 0.004), perineural invasion (HR: 1.67, 95% CI: 1.22–2.27, *p* = 0.001), vascular invasion (HR: 1.66, 95% CI: 1.19–2.31, *p* = 0.003), and not undergoing or completing adjuvant chemotherapy (HR: 3.06, 95% CI: 2.05–4.58, *p* < 0.001) were other risk factors. For stage III RFS, right side tumor location was an independent risk factor (HR: 1.40, 95% CI: 1.03–1.91, *p* = 0.030, **Table 6**).

## Discussion

Instant management is needed for acute complete OCC (COCC). Recommended treatments are different according to tumor location (1, 2, 4–6, 18, 19). According to 2017 WSES guidelines on colon cancer obstruction, for right COCC, right colectomy with primary anastomosis is a preferred treatment option (18). However, for left COCC, colectomy with or without primary anastomosis, endoscopic colonic stent insertion by self-expanding metallic stents (SEMS), tube decompression, and stoma formation are all possible options. The present study only analyzed patients with incomplete obstruction who did not require emergent management. All patients had undergone elective colectomy with primary anastomosis. Overall postoperative complications and CDC grade 3 or higher complications were more frequent in IOCC than in NOCC. However, there was no mortality case. There were no significant differences in readmission within 30 days or length of hospital stays between the two groups. This meant that there was no significant increase in hospitalization, readmission, or mortality, although the IOCC group had a high rate of postoperative complications. Moreover, anastomosis leakage rates did not differ significantly between the two groups. Therefore, for IOCC, regardless of the tumor location, colectomy with primary anastomosis can be an appropriate treatment procedure without increasing mortality or severe complications caused by primary anastomosis such as anastomotic leakage.

Many studies have shown that obstructive colon cancer has a poor prognosis (3, 7–9). According to NCCN guidelines (20), obstruction in stage II colon cancer is a high-risk factor and an indication of adjuvant chemotherapy. In our study, stage II IOCC showed similar overall and recurrence-free survival curves to stage III NOCC, with stage III IOCC showing a significantly lower survival rate. Furthermore, incomplete colon obstruction at all stages was an independent risk factor for worse OS and RFS in multivariable analysis. Our study showed that incomplete obstruction was a definitive factor affecting prognosis. Patients with incomplete obstruction had worse prognoses in stages 2 and 3 than those without incomplete obstruction. However, since COCC patients were not included in this study, it was not confirmed whether COCC and IOCC had the same survival rate, making it difficult to say whether they should be regarded as the same risk factors. Moreover, in this study, we could not find out whether chemotherapy helped improve survival of patients with stage II IOCC because we did not analyze the difference in survival following chemotherapy administration for stage II IOCC without other risk factors due to the small sample size. Therefore, further study on this is needed. Nevertheless, results of our study confirmed that the prognosis of IOCC was poor. This is meaningful in that it can provide more accurate information on prognosis of IOCC patients.

We also sub-analyzed characteristics and survivals according to tumor location to find any differences by tumor side. Some studies have investigated whether there are oncologic differences between left-sided and right-sided obstructive colon cancer. Frago et al. have compared proximal and distal obstructive colon cancer and reported that tumor location does not influence the prognosis after curative surgery (13). However, Mege et al. have reported that patients with right-sided OCC have significantly lower 5-year OS and RFS than those with left-sided OCC (OS: 43% for right-sided OCC vs. 53% for left-sided OCC, *p* < 0.0001; RFS: 36% for right-sided OCC vs. 46% for left-sided OCC, *p* = 0.001) (11). However, the present study did not show a significant difference in OS or RFS by tumor location for stage II. On the other hand, for stage III colon cancer patients, right-sided IOCC showed significantly lower survival than left-sided IOCC. In multivariable analysis, right side tumor location was also an independent risk factor for RFS, but not OS, in stage III colon cancer. These results indicate that the location of the tumor might have a sufficient impact on the survival of IOCC patients with lymph node metastasis, showing worse outcomes for right-sided tumors than for left-sided tumors.

This study has some limitations. First, there may be bias in the analysis because there were relatively fewer IOCC patients (*n* = 405) included compared to NOCC patients (*n* = 1599). Second, as mentioned above, it did not include complete obstruction, making it hard to know differences in survival between complete obstruction and incomplete obstruction. Liu et al. (21) have compared short-term and long-term outcomes between incomplete and complete left-sided malignant obstruction cases. There was no significant difference in survival between IOCC and COCC groups in their study. However, since their study only included left-sided tumors, it was difficult to assess whether survival was different for those with right-sided colon cancer. Further studies that compare survivals between right-sided IOCC and COCC are needed. Third, since our hospital is a tertiary hospital, most patients who visited our hospital usually received colonoscopy at a primary or secondary healthcare hospital. In addition, various physicians performed colonoscopy. Due to these characteristics, it is challenging to evaluate obstruction objectively as many physicians who have performed colonoscopy have different abilities. Their ability to pass through obstruction might also be different. Moreover, the degree of difficulty of performing colonoscopy may vary depending on the location of the tumor, such as hepatic flexure colon cancer, which may be a limitation in evaluating the oncologic outcomes according to the tumor location. However, the strength of this study was that it only included non-emergent obstructive colon cancer cases. Thus, morbidities in an emergent situation would not affect survival (4–6).

In conclusion, patients with IOCC showed significantly worse survival outcomes than those with NOCC. In addition, stage III right-sided IOCC patients showed significantly worse oncologic outcomes than left-sided colon cancer patients or right-sided NOCC patients. Based on these results, we can inform patients that the prognosis is different depending on the presence of incomplete obstruction and the location of the tumor, even in the same stage. However, further studies are needed to determine whether chemotherapy can help improve the prognosis of patients with IOCC, especially stage II.

## Data Availability Statement

The raw data supporting the conclusions of this article will be made available by the authors, without undue reservation.

## Ethics Statement

The studies involving human participants were reviewed and approved by the Institutional Review Board of Samsung Medical Center. Written informed consent for participation was not required for this study in accordance with the national legislation and the institutional requirements.

## Author Contributions

JL and WL contributed to the conception and design of the study. JL, WL, JH, SY, HK, YC, YP, and JS organized the database. JL performed the statistical analysis. JL and WL wrote the first draft of the manuscript. All authors contributed to manuscript revision, read, and approved the submitted version.

## Conflict of Interest

The authors declare that the research was conducted in the absence of any commercial or financial relationships that could be construed as a potential conflict of interest.

## Publisher’s Note

All claims expressed in this article are solely those of the authors and do not necessarily represent those of their affiliated organizations, or those of the publisher, the editors and the reviewers. Any product that may be evaluated in this article, or claim that may be made by its manufacturer, is not guaranteed or endorsed by the publisher.
